# Tumor-Associated Macrophages Provide Significant Prognostic Information in Urothelial Bladder Cancer

**DOI:** 10.1371/journal.pone.0133552

**Published:** 2015-07-21

**Authors:** Minna M. Boström, Heikki Irjala, Tuomas Mirtti, Pekka Taimen, Tommi Kauko, Annika Ålgars, Sirpa Jalkanen, Peter J. Boström

**Affiliations:** 1 MediCity Research Laboratory, Department of Medical Microbiology and Immunology, University of Turku, Turku, Finland; 2 Department of Otorhinolaryngology—Head and Neck Surgery, Turku University Hospital, Turku, Finland; 3 Department of Pathology, Helsinki University Hospital (HUSLAB) and Institute for Molecular Medicine Finland (FIMM), University of Helsinki, Helsinki, Finland; 4 Department of Pathology, Turku University Hospital, Turku, Finland; 5 Department of Biostatistics, University of Turku, Turku, Finland; 6 Department of Oncology and Radiotherapy, Turku University Hospital, Turku, Finland; 7 Department of Urology, Turku University Hospital, Turku, Finland; Louisiana State University Health Sciences center, UNITED STATES

## Abstract

Inflammation is an important feature of carcinogenesis. Tumor-associated macrophages (TAMs) can be associated with either poor or improved prognosis, depending on their properties and polarization. Current knowledge of the prognostic significance of TAMs in bladder cancer is limited and was investigated in this study. We analyzed 184 urothelial bladder cancer patients undergoing transurethral resection of a bladder tumor or radical cystectomy. CD68 (pan-macrophage marker), MAC387 (polarized towards type 1 macrophages), and CLEVER-1/Stabilin-1 (type 2 macrophages and lymphatic/blood vessels) were detected immunohistochemically. The median follow-up time was 6.0 years. High macrophage counts associated with a higher pT category and grade. Among patients undergoing transurethral resection, all studied markers apart from CLEVER-1/Stabilin-1 were associated with increased risk of progression and poorer disease-specific and overall survival in univariate analyses. High levels of two macrophage markers (CD68/MAC387^+/+^ or CD68/CLEVER-1^+/+^ groups) had an independent prognostic role after transurethral resection in multivariate analyses. In the cystectomy cohort, MAC387, alone and in combination with CD68, was associated with poorer survival in univariate analyses, but none of the markers were independent predictors of outcome in multivariate analyses. In conclusion, this study demonstrates that macrophage phenotypes provide significant independent prognostic information, particularly in bladder cancers undergoing transurethral resection.

## Introduction

The association between carcinogenesis and inflammation is generally accepted, and tumor-promoting inflammation is one of the hallmarks of cancer [1]. Inflammatory cells, chemokines, and cytokines are present in tumors from the earliest stages and are indispensable participants in the neoplastic process [2, 3]. Tumor-associated macrophages (TAMs) derived from peripheral blood monocytes and recruited by chemokines are a major component of the leukocyte infiltrate in tumors. Plasticity and diversity are universal features of mononuclear phagocytes, which can have either a protective or a tumor-promoting role, depending on microenvironmental signals [4]. TAMs are generally oriented towards promoting tumor growth and angiogenesis, suppressing adaptive immunity, and they have an important role in tumor cell migration, invasion, and metastasis. However, macrophages can also eliminate tumor cells and are therefore sometimes associated with better disease prognosis [2, 5]. Despite progress in understanding the interplay between inflammation and cancer, important questions remain unanswered. Cancer-related inflammation differs among tumor types and it is important to define which components are specific to particular tissues and tumors. It will be important to find the optimal stimuli to change a tumor-promoting microenvironment to a tumor-inhibiting one, and to understand the signaling mechanisms involved.

Bladder cancer (BC) is a heterogeneous disease. Non-invasive, well-differentiated tumors have a relatively indolent natural history, but poorly differentiated tumors are prone to invade and metastasize. In Western countries, BC is the fourth most common cancer in men [6]. Transurethral resection of the bladder tumor (TUR-BT) is used to diagnose and stage all tumors. While non-muscle-invasive BCs (NMIBC) may not require additional treatment, radical cystectomy (RC) with or without perioperative chemotherapy is considered the gold standard in the treatment of invasive BC and in NMIBC failing intravesical therapy.

Only limited data are available on the prognostic value of TAMs or their phenotype in BC, and most studies have concentrated on investigating TAMs in response to Bacillus Calmette-Guerin (BCG) immunotherapy. The aim of this study was to investigate the relationship between TAMs and clinicopathological variables in the entire spectrum of BC and to study the prognostic role of TAMs in BC after TUR-BT and RC using immunohistochemical methods.

## Materials and Methods

### Patients

The study protocol was approved by the Research Ethics Board of the Hospital District of Southwest Finland (1.8.2006/301). Written consent was obtained from the participants. Consecutive BC patients undergoing TUR-BT (in 2000–2004) or RC (in 1985–2005) at Turku University Hospital were included in the study. After exclusion, 184 patients were included in the study. The exclusion criteria were the following: 1) non-urothelial BC, 2) any intravesical instillations (BCG or chemotherapy) or systemic chemotherapy prior to study inclusion, and 3) insufficient tissue material available for histological re-review and immunohistochemistry.

TUR-BT was performed using standard techniques, and immediate single chemotherapy was instilled in 22% (20/92) of the patients. BCG or intravesical chemotherapy instillations were administered to T1 tumors and tumors with frequent recurrences. Specifically, intravesical therapy was administered to 39% of the patients (36/92). RC was performed for T1 tumors not responding to BCG and all ≥T2 tumors if fit for surgery. RC included removal of the bladder, prostate, and seminal vesicle in men and the uterus, ovaries, and anterior vaginal wall in females. Lymphadenectomy was not uniformly performed, but macroscopically suspicious lymph nodes were removed throughout the study period and limited pelvic lymph node dissection was carried out from 1995 onwards. Adjuvant therapy was not administered after RC and systemic chemotherapy was offered only at the time of metastatic progression during follow-up. After RC, patients were followed every 3 months for the first year and semiannually thereafter.

A detailed clinicopathological database was assembled retrospectively, including information regarding the patient and tumor characteristics, as well as details of the treatment and follow-up. Histological samples were re-reviewed for histology, differentiation grade, and stage by an expert uropathologist. The tumors were graded according to both the World Health Organization (WHO) 1973 and the WHO/International Society of Urinary Pathologist (ISUP) 2004 classifications and staged according to 2010 TNM staging [7, 8].

### Immunohistochemistry and scoring

For each case, the most representative formalin-fixed, paraffin-embedded tissue block was selected for analysis. Sections (5 μm thick) were deparaffinized with xylene and rehydrated with a graded alcohol series. The primary antibodies used were mouse monoclonal IgG_1_ anti-CD68 (KP1) (concentration 1:5; ab845, Abcam, U.K.) and mouse monoclonal IgG_1_ anti-MAC387 (concentration 1:500; ab22506, Abcam, U.K.), which detects the myelomonocytic L1 molecule calprotectin. CLEVER-1/Stabilin-1^+^ (common lymphatic endothelial and vascular endothelial receptor-1, also known as STAB1 and FEEL-1) type 2 macrophages and vessels were detected with the rat IgG 2–7 antibody (concentration 1:5) [9, 10]. The antibodies 3G6 (mouse IgG_1_ antibody against chicken T cells) [11] and MEL-14 [rat IgG_2a_ antibody against mouse L-selectin (CD62L)] (Exbio, Czech Republic) were used as negative controls. The primary immunoreaction was performed with using the mouse/rat Vectastain Elite ABC Kit (Vector Laboratories). Sections for anti-CD68-and MAC387 stainings were heat pre-treated in citrate acid (0.01M, pH 6.0) in a 97°C water bath for 20 min. Antigen retrieval for Clever-1/Stabilin-1-stained sections was performed with proteinase K (DAKO) (10min at 37°C) and the slides were washed three times with PBS after the pre-treatment. Endogenous peroxidase was blocked with 0.1% H_2_O_2_ for 30 min. Non-specific sites were blocked with horse (CD68 and MAC387) or rabbit (Clever-1/Stabilin-1) normal serum at room temperature for 20 min. Sections were incubated with primary antibodies overnight at 4°C and then treated with biotinylated secondary antibody solution according to the manufacturer’s instructions. After washing with PBS, Vectastain Elite ABC Reagent was added (30 min at room temperature), the slides were washed, and immunoreactions were detected using 3,3′-diaminobenzidine as a substrate. Slides were counterstained with hematoxylin, dehydrated, re-fixed in xylene, mounted with distyrene plasticizer xylene (DPX).

The whole tumor and surrounding peritumoral area were screened by light microscopy. The numbers of CD68^+^ macrophages, MAC387^+^ macrophages, and CLEVER-1/Stabilin-1^+^ macrophages and vessels were scored from three hotspots (areas with the most macrophages by eye) intratumorally and peritumorally with a 0.0625 mm^2^ grid using 40× magnification when scoring macrophages and 20× when scoring lymphatic/blood vessels. CLEVER-1/Stabilin-1^+^ is not normally present on flat-walled venules, but it is aberrantly induced on tumor vasculature [12]. The scoring was performed independently by two observers (MB and HI) blinded to the clinical information. Cases with inadequate quality of immunohistochemical staining or tumor morphology were excluded from further statistical analyses. The mean numbers of macrophages and vessels in three hotspots were calculated within one high-powered field. In the case of discordance, the sections were jointly reviewed to reach a consensus. MAC387^+^ tumor cells were graded semi-quantitatively into four categories, from–(negative) to +++ (abundant). The number of positive macrophages for each antibody was also graded semi-quantitatively into four categories, from—to +++. As the semi-quantitative scoring and the quantitative total count analysis associated significantly in all immunoanalyses (p < 0.001), detailed statistical analyses, including associations between immunosignals and survival, were carried out using the values obtained from the quantitative scoring system. Patient subgroups were created by combining two macrophage markers. The macrophage markers tested were divided into either low (-) or high (+) groups according to the mean value of the population. The following subgroups were generated: 1) low levels of both macrophage subtypes (^-/-^), 2) high level of either macrophage subtype, low level of the other macrophage subtype (^+/-^), and 3) high levels of both macrophage subtypes (^+/+^).

### Statistical analyses

The associations between CD68, MAC387, and CLEVER-1/Stabilin-1 expression and clinicopathological variables were evaluated with the Mann-Whitney U test and the Kruskal-Wallis test. Patient characteristics and the results of tests of normality for continuous variables in the total study cohort are shown in S1 Table. The Kaplan-Meier method, log-rank testing, and Cox proportional hazards regression models were used to analyze the associations between immunosignals and outcome. For Kaplan-Meier analyses, the mean macrophage and vessel counts were dichotomized according to the mean number. In the Cox proportional hazards regression models, the markers were evaluated as continuous variables. Outcome measures included disease-specific and overall survival (DSS and OS) in the RC population and DSS, OS, recurrence, and progression-free survival (PFS) in the TUR-BT population. The survival time was calculated from the date of surgery to the date of the last follow-up or death. Any death due to BC or with metastatic BC was defined as cancer-specific mortality. Recurrence was defined in the TUR-BT population as a histologically confirmed new tumor after a tumor-free period, and progression was defined as when a recurrence had a higher grade or a more advanced pT category than the primary tumor, or if the patient underwent RC for recurrence. All statistical tests were two-sided and p-values ≤0.05 were considered to be statistically significant. Statistical analyses were performed with the SPSS 20 (IBM) and SAS System for Windows, version 9.3 (SAS Institute Inc., Cary, NC, USA).

## Results

### Clinicopathological characteristics of the study population

The baseline clinicopathological characteristics of the patient cohort are presented in Table 1. Most of the patients had NMIBC (pTa, pTcis, or pT1) (88% of the TUR-BT population and 36% of the RC population). The number of tumors was recorded in the TUR-BT population and of those patients, 63% had a single tumor. At the end of the follow-up period, 36% of the patients in the TUR-BT population and 28% of the patients in the RC population were alive. The median follow-up time was 6.9 and 4.2 years in the TUR-BT and RC populations, respectively.

**Table 1:** Baseline clinicopathological characteristics (n = 184).

Characteristic		TUR-BT^1^ n (%)	RC^2^ n (%)
Gender	Male	69 (75)	74 (80)
Age	Years, median (range)	71 (34–92)	65 (38–78)
Smoking	Never	34 (37)	43 (47)
	Current	24 (26)	39 (33)
	Former	11 (12)	15 (16)
	Unknown	22 (24)	4 (4)
Grade	Low	60 (55)	19 (21)
	High	32 (35)	73 (79)
pT category	pTa, pTcis, pT1	81 (88)	33 (36)
	pT2	11 (12)	24 (26)
	pT3	NA	23 (25)
	pT4	NA	12 (13)
Outcome	Alive	33 (36)	26 (28)
	Dead, bladder cancer	22 (24)	39 (42)
	Dead, other reason	37 (40)	17 (19)
	Lost for follow-up	22 (24)	10 (11)
Follow-up time	Months, median (range)	6.9 (0–14.3)	4.2 (0–17.8)

^1^ TUR-BT; Transurethral resection of bladder tumor

^2^ RC; Radical cystectomy.

### TUR-BT tumors have lower TAM counts than tumors from RC

**Fig 1:**
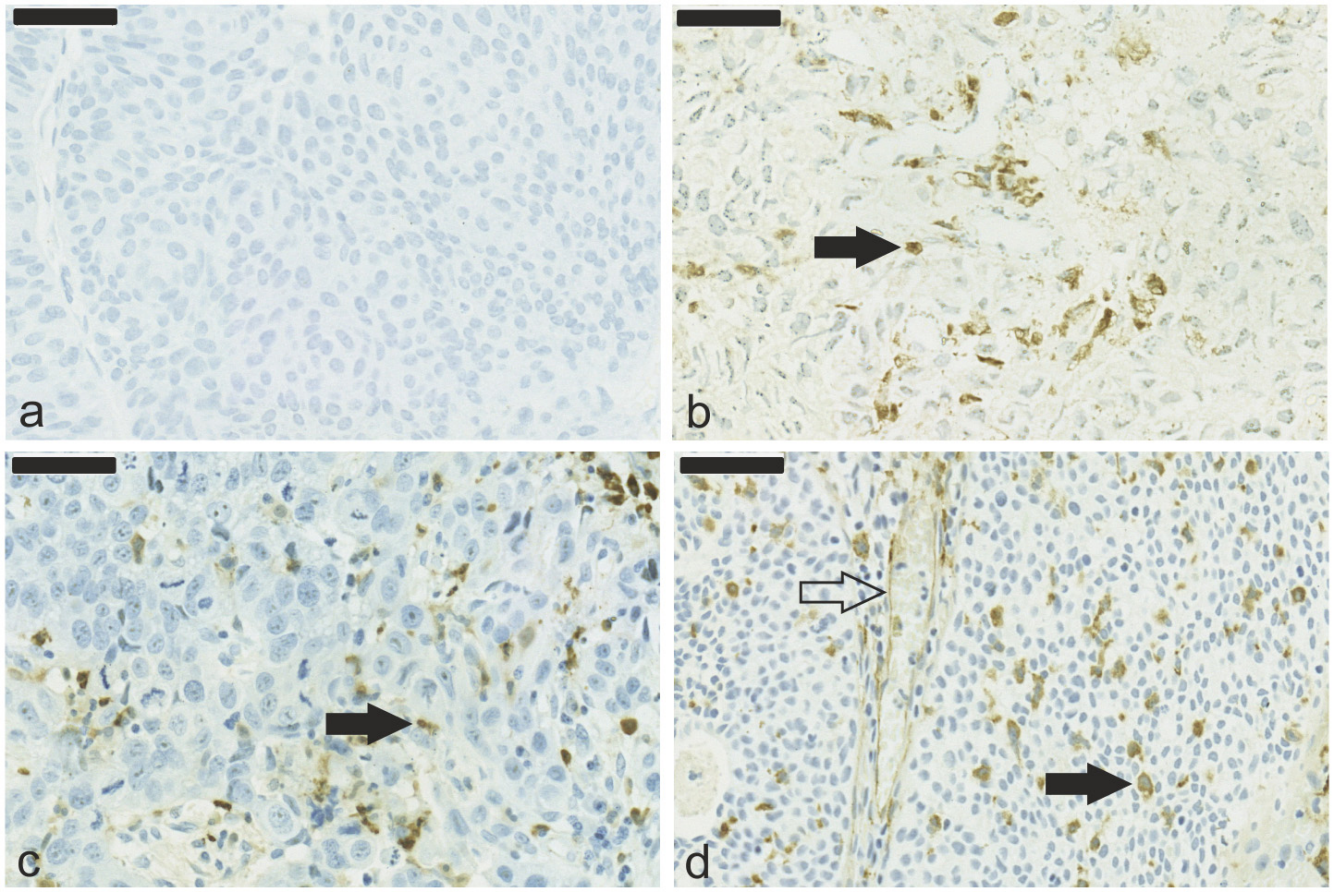
Immunohistological detection of macrophages in bladder cancer. TUR-BT specimens stained with a negative control antibody (a); intratumoral CD68^+^ macrophages (b), MAC387^+^ macrophages (c), and CLEVER-1/Stabilin-1^+^ macrophages and vessels (d). Filled arrows indicate positively stained macrophages, and unfilled arrows indicate CLEVER-1/Stabilin-1^+^ vessels. Magnification 40×, scale bar 100 μm.

Table 2a shows TUR-BT and RC patients dichotomized according to the mean number of macrophages and vessels, and representative examples of the staining patterns with different markers are shown in Fig 1.

**Table 2:** Macrophage and lymphatic/blood vessel counts in the TUR-BT and RC cohorts. Data represent the mean number of positive cells from three hotspots (one high-powered field per hotspot). Counts are represented as groups dichotomized according to the mean macrophage count. a, macrophage and vessel counts. b, cell counts according to the expression of two markers. Here, CLEVER-1 staining refers to expression in macrophages, not vessels.

a.			b.		
Marker	Number of macrophages	Number of patients (%)	Groups	TUR-BT No (%)	Radical cystectomy No (%)
CD68			CD68/MAC387		
TUR-BT	0–18	61 (66)	CD68/MAC387^-/-^	57 (62)	19 (39)
	19–81	31 (34)	CD68/MAC387^-/+^	25 (25)	17 (35)
Radical cystectomy	0–30	34 (60)	CD68/MAC387^+/+^	12 (13)	13 (26)
	31–102	23 (40)	CD68/CLEVER-1		
MAC387			CD68/CLEVER-1^-/-^	22 (24)	28 (51)
TUR-BT	0–19	62 (67)	CD68/CLEVER-1^-/+^	53 (58)	20 (36)
	20–135	30 (32)	CD68/CLEVER-1^+/+^	17 (19)	7 (13)
Radical cystectomy	0–34	41 (65)	MAC387/CLEVER-1		
	35–197	22 (35)	MAC387/CLEVER-1^-/-^	23 (25)	23 (43)
CLEVER-1 macrophages			MAC387/CLEVER-1^-/+^	50 (54)	24 (45)
TUR-BT	0–26	56 (61)	MAC387/CLEVER-1^+/+^	19 (21)	6 (11)
	27–73	36 (39)			
Radical cystectomy	0–11	43 (60)			
	12–41	29 (40)			
CLEVER-1vessels					
TUR-BT	0–8	56 (61)			
	9–22	36 (39)			
Radical cystectomy	0–1	42 (65)			
	2–6	23 (35)			

^-/-^; Macrophage counts lower than the mean value of both markers

^-/+^; Macrophage count lower than the mean value of one marker and higher that the mean value of the other.

^+/+^; Macrophage count higher than the mean values of both markers.

The mean number of intratumoral CD68^+^ macrophages was 18 per field in the TUR-BT population [standard deviation (SD) ± 16] and 30 in the RC population (SD ± 26). For MAC387, the mean macrophage counts were 19 (SD ± 24) and 34 (SD ± 38) in the TUR-BT and RC populations, respectively. The highest CLEVER-1/Stabilin-1^+^ macrophage count was 73 in the TUR-BT group and 41 in the RC group, and the mean numbers were 26 (SD ± 13) and 11 (SD ± 10), respectively. CLEVER-1/Stabilin^+^ lymphatic/blood vessel density ranged from 0 to 22 positive cells per field with a mean value of 8 in the TUR-BT population (SD ± 5) and 1 in the RC population (SD ± 1). The marker counts in the TUR-BT and RC cohorts are shown in S1 Fig Cell counts according to the expression of two macrophage markers are shown in Table 2b.

### High CD68^+^, high MAC387^+^, and low CLEVER-1/Stabilin-1^+^ macrophage counts are associated with conventional features of high risk in BC

**Fig 2:**
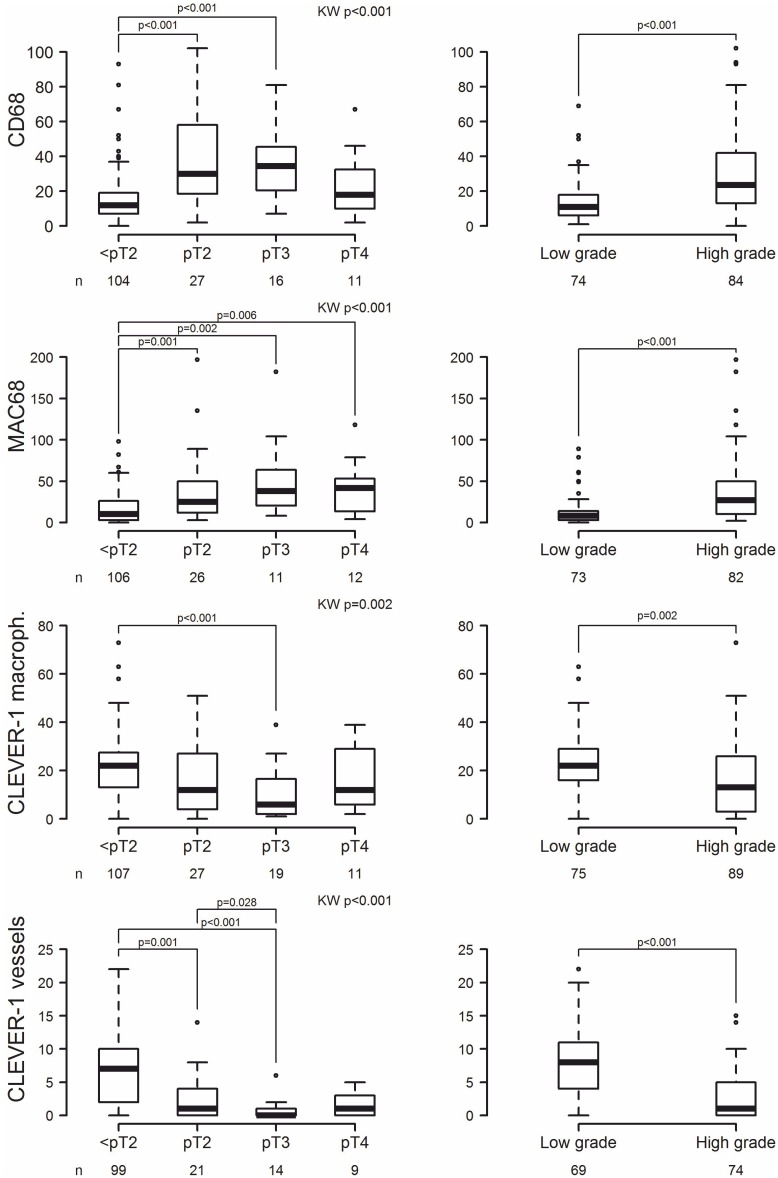
Group differences between pT category and tumor grade and CD68, MAC387, and CLEVER-1 expression. The Mann-Whitney U test was used for pair-wise comparisons in analyses of tumor grade and Kruskal-Wallis rank-sum testing (KW) was used for comparison across the four pT categories. The bottom and top edges of the box indicate the intra-quartile range (IQR), the line inside the box indicates the median value, the whiskers that extend from each box indicate the range of values that are outside of the intra-quartile range but closer than or equal to 1.5 times the IQR, and any points that are at a distance of more than 1.5 times the IQR from the box are considered to be outliers [circles indicate the mild outliers (more than 1.5 times the IQR), and asterisks extreme outliers (more than 3 times the IQR)].

Associations between tumor grade and pT category and the expression of CD68, MAC387, and CLEVER-1/Stabilin-1 are shown in Fig 2 (all p-values <0.05). High numbers of CD68^+^ macrophages and MAC387^+^ macrophages were associated with a higher pT category and tumor grade. In contrast, lower CLEVER-1/Stabilin-1^+^ macrophage/vessel counts were associated with a higher pT category and grade. Females had higher numbers of CLEVER-1/Stabilin-1^+^ macrophages (p = 0.003, Mann-Whitney U test, not shown). There were no significant associations between cell counts and smoking status or age.

### TAM counts predict the risk of progression after TUR-BT

#### TUR-BT population

**Fig 3:**
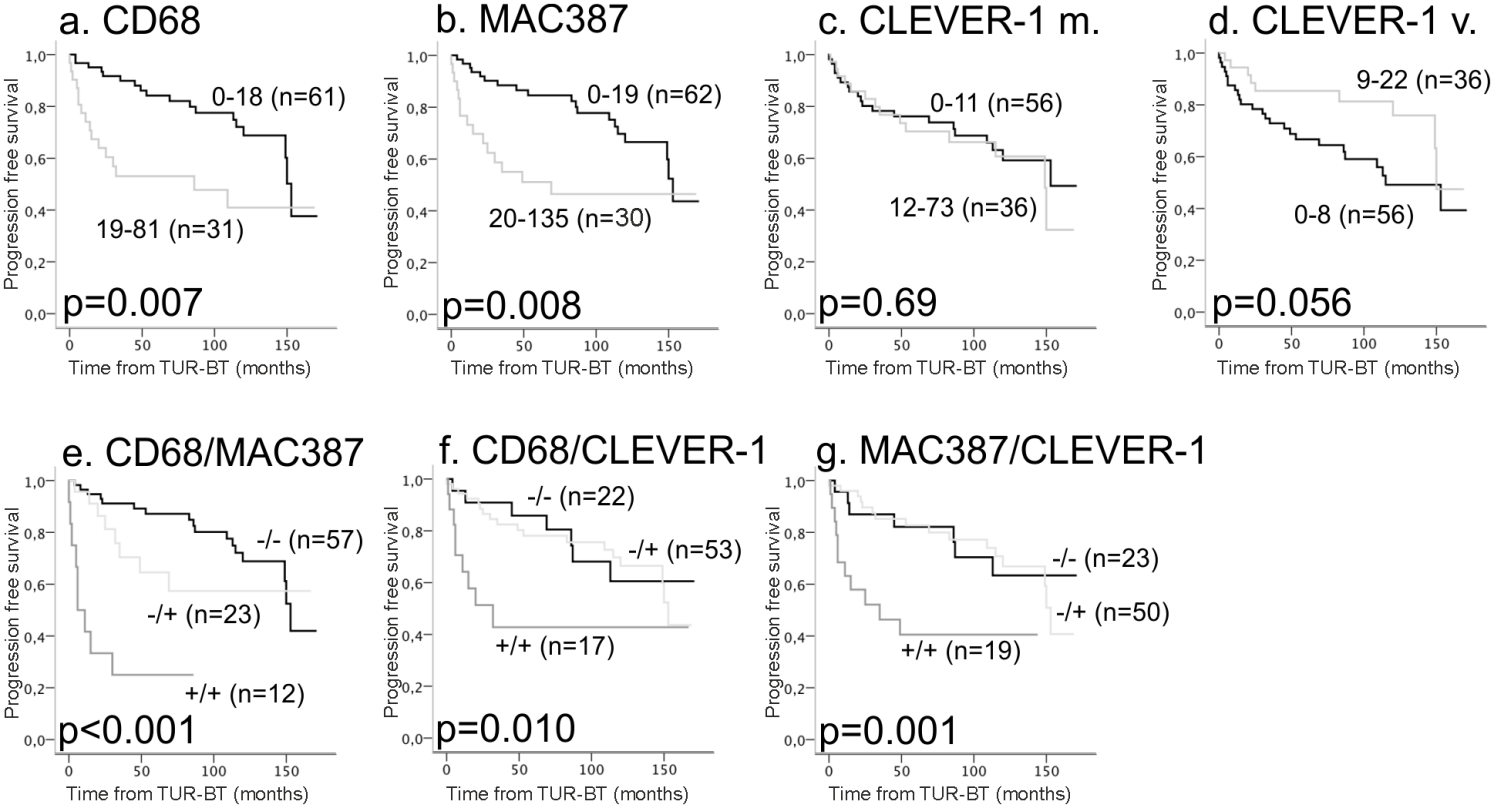
Kaplan-Meier estimates for progression in the TUR-BT population. The effect of CD68^+^ macrophages, MAC387^+^ macrophages, and CLEVER-1/Stabilin-1^+^ macrophages/vessels on progression in the TUR-BT population (a-d). Patients were divided into groups according to the expression of two macrophage markers, and associations with progression were determined (e-f).

Kaplan-Meier estimates evaluating the relationships between various macrophage populations and PFS in the TUR-BT population are shown in Fig 3. High numbers of CD68^+^ macrophages and MAC387^+^ macrophages were significantly associated with risk of progression (Fig 3a and 3b) (p-values 0.007 and 0.008, respectively). The CLEVER-1/Stabilin-1^+^ macrophage count did not affect the PFS (p = 0.69) (Fig 3c). A trend (p = 0.056) towards higher progression risk was observed with a lower CLEVER-1/Stabilin-1^+^ vessel count (Fig 3d). The associations between combinations of macrophage markers and PFS in the TUR-BT population are shown in Fig 3e and 3g. All patient groups with high macrophage numbers defined by two different macrophage markers (i.e., double high; CD68/MAC387^+/+^, CD68/CLEVER-1^+/+^, and MAC387/CLEVER-1^+/+^) had a shorter PFS compared to the other groups. When CD68 and MAC387 were analyzed together, patients with low levels of macrophages had the longest PFS. When CLEVER-1/Stabilin-1 positivity was analyzed together with CD68 or MAC387, there was no difference in the PFS between patients whose tumors were low for both markers compared to patients in which either one of the markers was high. Kaplan-Meier estimates for DSS and OS in the TUR-BT population showed similar results as for PFS except for CLEVER-1, where a higher vessel count was associated with worse survival (S2 and S3 Figs). By contrast, there were no associations observed between the recurrence risk and the tested markers alone, or in combination (S4 Fig).

Univariate and multivariate Cox proportional hazards regression models of factors affecting OS in the TUR-BT population are presented in Table 3. In the univariate analysis, established risk factors (high tumor grade, advanced pT category, higher age, and a high number of tumors) significantly associated with shorter OS. Numbers of CD68^+^ macrophages [hazard ratio (HR) 1.031 and 95% confidence interval (CI) 1.016–1.046; p<0.001] and MAC387^+^ macrophages (HR 1.016 and 95% CI 1.006–1.027; p = 0.002) significantly associated with OS in a univariate Cox regression model; however, these associations failed to remain significant in multivariate analyses. CLEVER-1/Stabilin-1^+^ macrophage/vessel counts did not predict survival. When data from two macrophage markers was combined, all patients who had high expression of both markers had higher mortality in univariate analysis than other groups. The CD68/MAC387^+/+^ and CD68/CLEVER-1^+/+^ groups remained to have significant associations with survival in multivariate analyses (CD68/MAC387^+/+^: HR 3.5 and 95% CI 1.1–11; p = 0.036, and CD68/CLEVER-1^+/+^: HR 3.8 and 95% CI 1.4–10, p = 0.008).

**Table 3:** Univariate and multivariate Cox proportional hazards regression analysis of factors affecting OS in the TUR-BT population.

	Univariate			Multivariate		
Variable	HR	95% CI	p-value	HR	95% CI	p-value
Grade						
Low	*REF*			*REF*		
High	3.2	1.9–5.5	<0.001*	1.2	0.58–2.4	0.65
pT category						
≤pT1	*REF*			*REF*		
≥pT2	3.8	1.9–7.6	<0.001*	1.6	0.64–3.8	0.32
Age	1.112	1.078–1.147	<0.001*	1.096	1.059–1.134	<0.001*
Number of tumors						
Single	*REF*			*REF*		
Multiple	1.7	1.0–3.0	0.044*	1.2	0.66–2.1	0.56
CD68	1.031	1.016–1.046	<0.001*	1.012	0.994–1.013	0.19
MAC387	1.016	1.006–1.027	0.002*	1.003^a^	0.989–1.018	0.65
CLEVER-1 macrophages	1.011	0.990–1.032	0.30	1.005^a^	0.985–1.026	0.62
CLEVER-1 vessels	0.958	0.901–1.019	0.17	1.015^a^	0.950–1.086	0.65
CD68/MAC387						
CD68/MAC387^-/-^	*REF*			*REF*		
CD68/MAC387^-/+^	1.8	0.99–3.3	0.054	1.6^a^	0.72–3.4	0.26
CD68/MAC387^+/+^	6.9	3.4–14	<0.001*	3.5^a^	1.1–11	0.036*
CD68/CLEVER-1						
CD68/CLEVER-1^-/-^	*REF*			*REF*		
CD68/CLEVER-1^-/+^	0.89	0.47–1.7	0.71	1.1^a^	0.55–2.3	0.77
CD68/CLEVER-1^+/+^	3.1	1.5–6.6	0.002*	3.8^a^	1.4–10	0.008*
MAC387/CLEVER-1						
MAC387/CLEVER-1^-/-^	REF			REF		
MAC387/CLEVER-1^-/+^	0.94	0.50–1.8	0.85	1.4^a^	0.71–2.8	0.34
MAC387/CLEVER-1^+/+^	2.9	1.4–5.9	0.004*	1.6^a^	0.66–3.7	0.32

* Significant p-value

^a^ Marker expression in multivariate analyses adjusted for grade, pT category, age, and tumor number. Each marker was analyzed in a separate multivariate analysis.

Univariate and multivariate Cox proportional hazards regression models of factors affecting DSS, PFS, and recurrence in the TUR-BT population are presented in S2–S4 Tables. Numbers of CD68^+^ and MAC387^+^ macrophages and of CLEVER-1/Stabilin-1^+^ vessels associated with DSS in univariate analyses but failed to remain significant in multivariate analyses. The double-high group and the CD68/MAC387^+/-^ group also significantly associated with DSS in univariate analyses but not in multivariate analyses (S2 Table). Individual markers and groups of two markers associated similarly with PFS (S4 Table).

#### RC population

**Fig 4:**
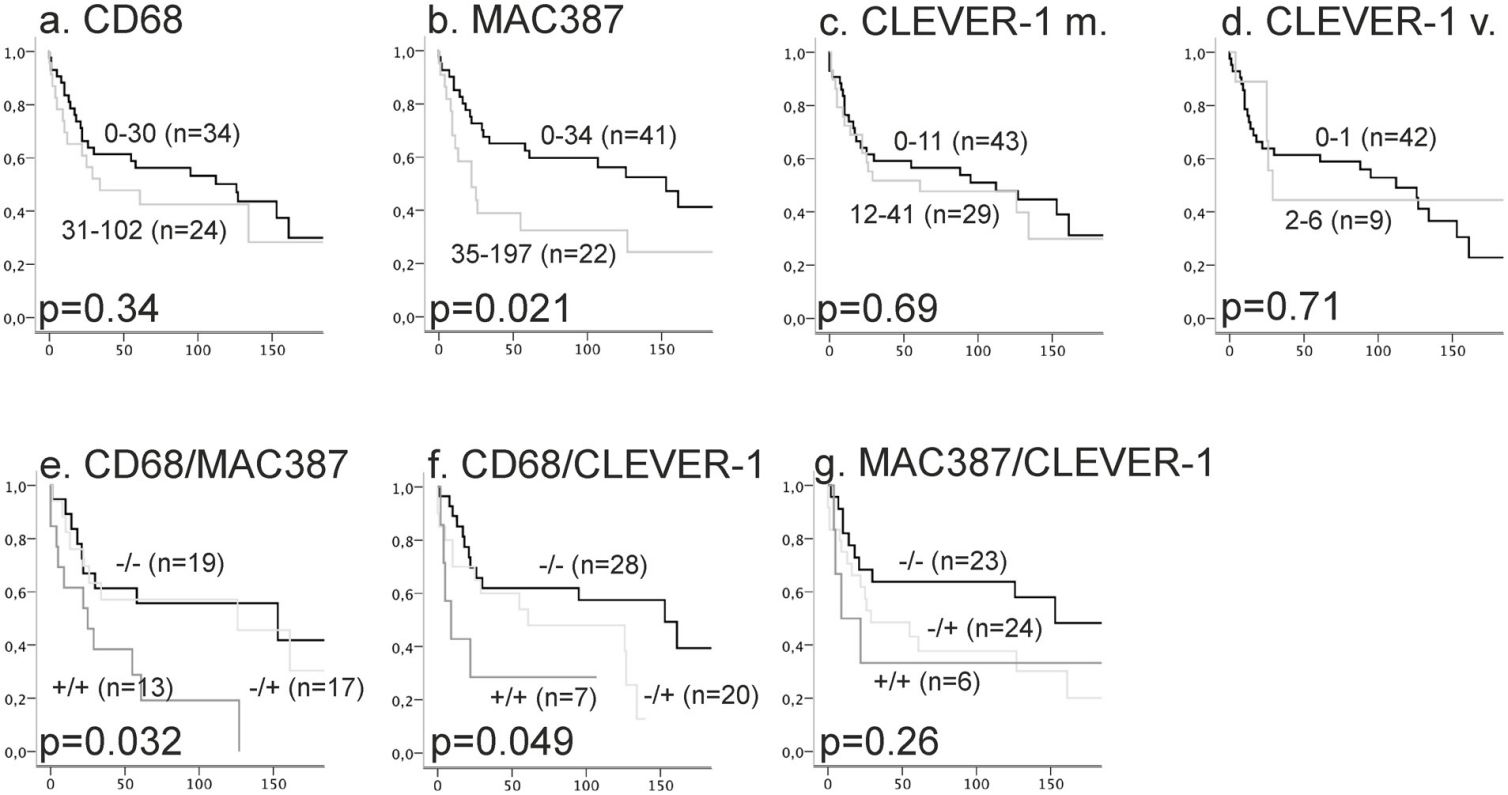
Kaplan-Meier estimates for OS in the RC population. The effect of CD68^+^ macrophages, MAC387^+^ macrophages, and CLEVER-1/Stabilin-1^+^ macrophages/vessels on the OS in the radical cystectomy population (a-d). The association between OS and the expression of two macrophage markers (e-f).

Kaplan-Meier estimates for OS in the RC population are presented in Fig 4. A high MAC387^+^ macrophage count associated with a greater risk of death (p = 0.021). Other markers did not associate with OS. When marker data were combined, tumors that had high levels of CD68^+^ macrophages and MAC387^+^ or CLEVER-1^+^ macrophages had a shorter OS (CD68/MAC387^+/+^ p = 0.032 and CD68/CLEVER-1^+/^ p = 0.049). When DSS was analyzed, the groups combining two macrophage markers predicted survival, as with OS (S5 Fig).

Univariate and multivariate Cox proportional hazards regression analyses of factors affecting OS in the RC population and the combined expression of two macrophage markers are shown in Table 4. Tumor grade and pT category associated significantly with OS. None of the macrophage markers associated significantly with OS in uni- or multivariate analyses. The CD68/MAC387^+/+^ double-high group associated significantly with OS in univariate analysis (HR 2.9 and 95% CI 1.2–7.2; p = 0.020). Cox proportional hazards regression analyses of factors affecting DSS are shown in S4 Table. MAC387 expression (HR 1.008 and 95% CI 1.001–1.016; p = 0.032) and CD68/MAC387^+/+^ dual expression (HR 3.5 and 95% CI 1.1–11; p = 0.029) associated significantly with DSS in univariate analysis, but none of the markers, alone or in combination, significantly predicted outcome in multivariate analyses.

**Table 4:** Univariate and multivariate Cox proportional hazards regression analysis of factors affecting OS in the RC population.

	Univariate			Multivariate		
Variable	HR	95% CI	p-value	HR	95% CI	p-value
Grade						
Low	*REF*			*REF*		
High	1.8	0.86–3.6	0.12	1.7	0.65–4.4	0.29
pT category						
≤pT1	*REF*			*REF*		
pT2	2.3	1.1–5.0	0.027*	2.4	0.95–6.3	0.065
pT3	4.7	2.2–10	<0.001*	4.3	1.6–11	0.003*
pT4	6.4	2.7–15	<0.001*	5.3	1.8–16	0.002*
Age	1.009	1.980–1.040	0.54	1.002	0.983–1.012	0.92
CD68	1.004	0.993–1.015	0.51	0.997	0.983–1.012	0.72
MAC387	1.006	0.999–1.013	0.070	1.002^a^	0.994–1.010	0.56
CLEVER-1 macrophages.	1.017	0.986–1.049	0.28	1.017^a^	0.987–1.049	0.27
CLEVER-1 vessels	1.052	0.768–1.442	0.75	1.149^a^	0.839–1.573	0.39
CD68/MAC387						
CD68/MAC387^-/-^	*REF*			*REF*		
CD68/MAC387^-/+^	1.2	0.48–3.0	0.70	1.1^a^	0.34–3.5	0.88
CD68/MAC387^+/+^	2.9	1.2–7.2	0.020*	2.1^a^	0.79–5.7	0.13
CD68/CLEVER-1						
CD68/CLEVER-1^-/-^	*REF*			*REF*		
CD68/CLEVER-1^-/+^	2.0	0.89–4.5	0.095	1.3^a^	0.53–3.0	0.60
CD68/CLEVER-1^+/+^	3.4	1.2–10	0.027	2.2^a^	0.69–6.8	0.18
MAC387/CLEVER-1						
MAC387/CLEVER-1^-/-^	*REF*			*REF*		
MAC387/CLEVER-1^-/+^	1.8	0.84–3.8	0.14	1.0^a^	0.44–2.4	0.95
MAC387/CLEVER-1^+/+^	2.0	0.63–6.4	0.24	2.6^a^	0.72–9.1	0.15

* Significant p-value

^a^ Marker expression in a multivariate analyses adjusted for grade, pT category, and age. Each marker was analyzed in a separate multivariate analysis.

## Discussion

In the present study, we demonstrate that the intratumoral macrophage density (CD68^+^ and MAC387^+^ macrophages) is significantly associated with conventional high-risk features including high grade and advanced T category in BC. Furthermore, a high macrophage count was significantly associated with risk of progression in the TUR-BT cohort and survival in the cystectomy cohort by univariate analyses. Although single macrophage markers were not significant predictors in multivariate analyses, combinations of two markers (CD68 with MAC387 or CLEVER-1) provided independent prognostic information in multivariate models in the TUR-BT cohort when factors affecting survival were analyzed. CLEVER-1/Stabilin-1^+^ macrophages were not associated with BC mortality, but a high CLEVER-1/Stabilin-1^+^ vessel count, by contrast, was associated with improved survival in univariate models in the TUR-BT cohort.

A high TAM density has been reported to be associated with poor prognosis in various malignancies, such as breast cancer, melanoma, colorectal cancer, and prostate cancer [13]. Hanada and co-workers demonstrated in a smaller study (23 MIBC and 40 NMIBC cases) that invasive tumors that had higher CD68^+^ macrophage counts and higher TAM counts were associated with higher rates of cystectomy, distant metastases, vascular invasion, and poorer survival [14]. Recently, Sjödahl and co-workers studied TAM counts in MIBC using tissue microarray (TMA) sections stained with anti-CD3 for T cells, anti-CD8 for cytotoxic T cells, anti-FOXP3 for regulatory T cells (Tregs), and two macrophage-specific markers, CD68 and CD163. They noted that the clinical stage combined with the tumor CD68/CD3 ratio could be a potential tool for prognostication [15]. However, we have noticed that macrophages appear in clusters within tumors, and thus using whole sections instead of TMAs is likely to be more accurate, as TMA cores represent only small portions of the entire specimen. Moreover, some of the patients in the study had received BCG instillations or neoadjuvant chemotherapy, which may have affected the results.

NMIBC tumors with high TAM counts have also been shown to have worse outcome and to be less responsive to BCG treatment [16–18]. Inflammatory processes in the bladder in response to BCG bacteria are being utilized to destruct tumor tissue. Cells of the urothelium respond to the treatment with an inflammatory cascade and release cytokines, e.g., interleukin (IL)-8 and tumor necrosis factor (TNF), and recruit neutrophils to destroy malignant cells [19]. In our study, patients who received intravesical installations (BCG or chemotherapy) were excluded due to the fact that these treatments change the inflammatory microenvironment of the tumors and thus could alter the results.

There are several challenges in BC care. The recurrence rate of NMIBC is high, and frequent procedures and intense follow-up results in BC being one of the most expensive cancers to treat [20]. Treatment options for advanced diseases are limited and novel therapies are needed, including new biomarkers to diagnose aggressive cases that require more intensive treatment. CD68, MAC387, and CLEVER-1/Stabilin-1, especially when used in combination, could potentially be used to identify aggressive cases among BC patients. These macrophages could also be the targets of novel treatments for advanced BC. Recently, PD-L1 inhibitors have demonstrated potential efficacy in metastatic BC, and more research in BC immunology is needed [21].

The antibody used most often for the detection of TAMs is the pan-macrophage marker CD68 and it has been shown to associate with poor survival in BC [14]. In this study, we also used MAC387 to detect different macrophage populations. An association between a high MAC387^+^ macrophage count and poor outcome has been reported in various tumors, such as breast cancer and cholangiocarcinoma [22–24]. To our best knowledge, MAC387 has been investigated only as a marker of squamous differentiation in BC [25, 26], and our work is the first to detect a relationship between MAC387^+^ macrophage density and poor outcome in BC patients. Both MAC387^+^ macrophages and tumor cells were evaluated, but the number of MAC387-positive tumor cells did not associate with survival (data not shown). MAC387 recognizes myelo-related protein (MRP) 14 and, to a lesser extent, the MRP8/MRP14 heterocomplex (calprotectin). MRP14 is a pro-inflammatory molecule expressed during acute inflammation on recently infiltrating monocytes/macrophages, whereas MRP8 is expressed on macrophages during chronic inflammation. Tissue macrophages have been shown to express a MRP8/MRP14 complex under some circumstances: e.g., at sites of chronic inflammation in rheumatoid arthritis, sarcoidosis, and tuberculosis, but not in normal tissue without any inflammation [27, 28]. Soulas and co-workers have considered MAC387^+^ cells to be pro-inflammatory and anti-tumoral M1-polarized macrophages [27]. However, this is not certain, and there is still a lack of a definitive surface marker for pro-inflammatory type 1 macrophages.

In addition to CD68 and MAC387, we used CLEVER-1/Stabilin-1 to detect type 2 activated macrophages. CLEVER-1/Stabilin-1 is a large scavenger receptor on a subset of immunosuppressive and protumoral type 2 macrophages with multiple functions [29, 30]. CLEVER-1/Stabilin-1 is also expressed in afferent and efferent lymphatic and sinusoidal endothelial cells [10]. CLEVER-1/Stabilin-1^+^ macrophage numbers were not associated with patient survival in BC, contrary to our previous studies in colorectal cancer [12]. Interestingly, there was a clear relationship between low CLEVER-1/Stabilin-1^+^ vessel count and increasing tumor stage and higher grade. Further, a high CLEVER-1/Stabilin-1^+^ vessel count was associated with better survival after RC in BC patients. The reason for CLEVER-1/Stabilin-1^+^ vessels acting in a protective fashion is unclear and further studies are warranted. To our best knowledge, MAC387^+^ macrophages and CLEVER-1/Stabilin-1 have not been studied in BC before.

In addition to the possible prognostic role of macrophage markers alone, we studied macrophage populations further by combining two markers. The CD68/MAC387^+/+^, CD68/CLEVER-1^+/+^ and MAC387/CLEVER-1^+/+^ groups had better OS in the TUR-BT population, and the CD68/MAC387^+/+^ and CD68/CLEVER-1^+/+^ groups were independent prognostic factors. As the markers detect different populations of TAMs (CD68 detects macrophages in general, while MAC387 detects type 1 polarized macrophages and CLEVER-1/Stabilin-1 detects type 2 macrophages), using these markers in combination may better identify tumors that were skewed either towards the M1 phenotype (CD68^+^/MAC387^+^ and MAC387^+^/CLEVER-1^-^) or the M2 type (CD68^+^/CLEVER-1^+^ and MAC387^-^/CLEVER-1^+^). However, in our study, the overall macrophage count had the most crucial role in the prognosis of BC, and surprisingly, this was the case regardless of whether the macrophages were predominantly type 1 or type 2.

The present study has the known limitations of retrospective cohort studies. There was a long follow-up time in the RC population, and some clinical practices may have changed during this time. During this period, the grading system for BC was changed and the 2004 WHO/ISUP classification was defined after the 1973 WHO classification. The 2004 WHO/ISUP classification was utilized in the entire cohort after re-review of all cases by an expert genitourinary pathologist. Furthermore, in RCs, the clinical practice of lymphadenectomy was not uniformly performed throughout the study period. On the other hand, the study cohort did not have the possible confounding factors seen in previous studies; e.g., all tumors were urothelial cancers and all cases with prior treatments were excluded. This is particularly important in the case of previous BCG instillations.

In conclusion, we have demonstrated a significant increase in TAM counts in more advanced tumors and in tumors with poor differentiation. A significant reverse association was noted between the CLEVER-1/Stabilin-1^+^ vessel count and tumor stage and grade. The association between high macrophage count and poor risk features most likely explains the observation that TAM density was prognostic for progression in the TUR-BT cohort and mortality in the cystectomy cohort in univariate analysis, but no independent prognostic information was noted. When information from two markers were combined, especially CD68 and MAC387 or CLEVER-1, the staining phenotype was a significant independent prognostic factor in the TUR-BT cohort for risk of progression. In addition to providing prognostic information, TAMs may be a potential treatment target in BC and are worthy of further study.

## Supporting Information

S1 Fig Marker counts in the TUR-BT and RC cohorts. The Mann-Whitney U test was used for pair-wise comparisons. The bottom and top edges of the box indicate the intra-quartile range (IQR), the line inside the box indicates the median value, and the whiskers that extend from each box indicate the range of values that are outside of the intra-quartile range but are closer than or equal to 1.5 times the IQR. Any points that are at a distance of more than 1.5 times the IQR from the box are considered to be outliers. Circles indicate the mild outliers (more than 1.5 times the IQR) and asterisks indicate extreme outliers (more than 3 times the IQR).
(TIF)

S2 Fig Kaplan-Meier estimates for DSS in the TUR-BT population. The effect of CD68^+^ macrophages, MAC387^+^ macrophages, and CLEVER-1/Stabilin-1^+^ macrophages/vessels on the DSS in the TUR-BT population (a-d). The association between DSS and the expression of two macrophage markers (e-f).
(TIF)

S3 Fig Kaplan-Meier estimates for OS on TUR-BT population. The effect of CD68^+^ macrophages, MAC387^+^ macrophages, and CLEVER-1/Stabilin-1^+^ macrophages/vessels on OS in the TUR-BT population (a-d). The association between OS and the expression of two macrophage markers (e-f).
(TIF)

S4 Fig Kaplan-Meier estimates for recurrence in the TUR-BT population. The effect of CD68^+^ macrophages, MAC387^+^ macrophages, and CLEVER-1/Stabilin-1^+^ macrophages/vessels on recurrence in the TUR-BT population (a-d). The association between recurrence and the expression of two macrophage markers (e-f)
(TIF)

S5 Fig Kaplan-Meier estimates for DSS on radical cystectomy population. The effect of CD68^+^ macrophages, MAC387^+^ macrophages, and CLEVER-1/Stabilin-1^+^ macrophages/vessels on DSS in the TUR-BT population (a-d). The association between DSS and the expression of two macrophage markers (e-f).
(TIF)

S1 Table Characteristics and normalities of scale variables in the whole study cohort. (DOCX)

S2 Table Univariate and multivariate Cox proportional hazards regression analysis of factors affecting DSS in the TUR-BT population. (DOCX)

S3 Table Univariate and multivariate Cox proportional hazards regression analysis of factors affecting recurrence in the TUR-BT population. (DOCX)

S4 Table Univariate and multivariate Cox proportional hazards regression analysis of factors affecting PFS in the TUR-BT population. (DOCX)

S5 Table Univariate and multivariate Cox proportional hazards regression analysis of factors affecting DSS in the RC population. (DOCX)
