# Supplementary figures and images for: Tumor-Associated Macrophages Provide Significant Prognostic Information in Urothelial Bladder Cancer
Source: PLoS One. 2015 Jul 21;10(7):e0133552. doi: 10.1371/journal.pone.0133552 (PMC4511010; doi:10.1371/journal.pone.0133552)

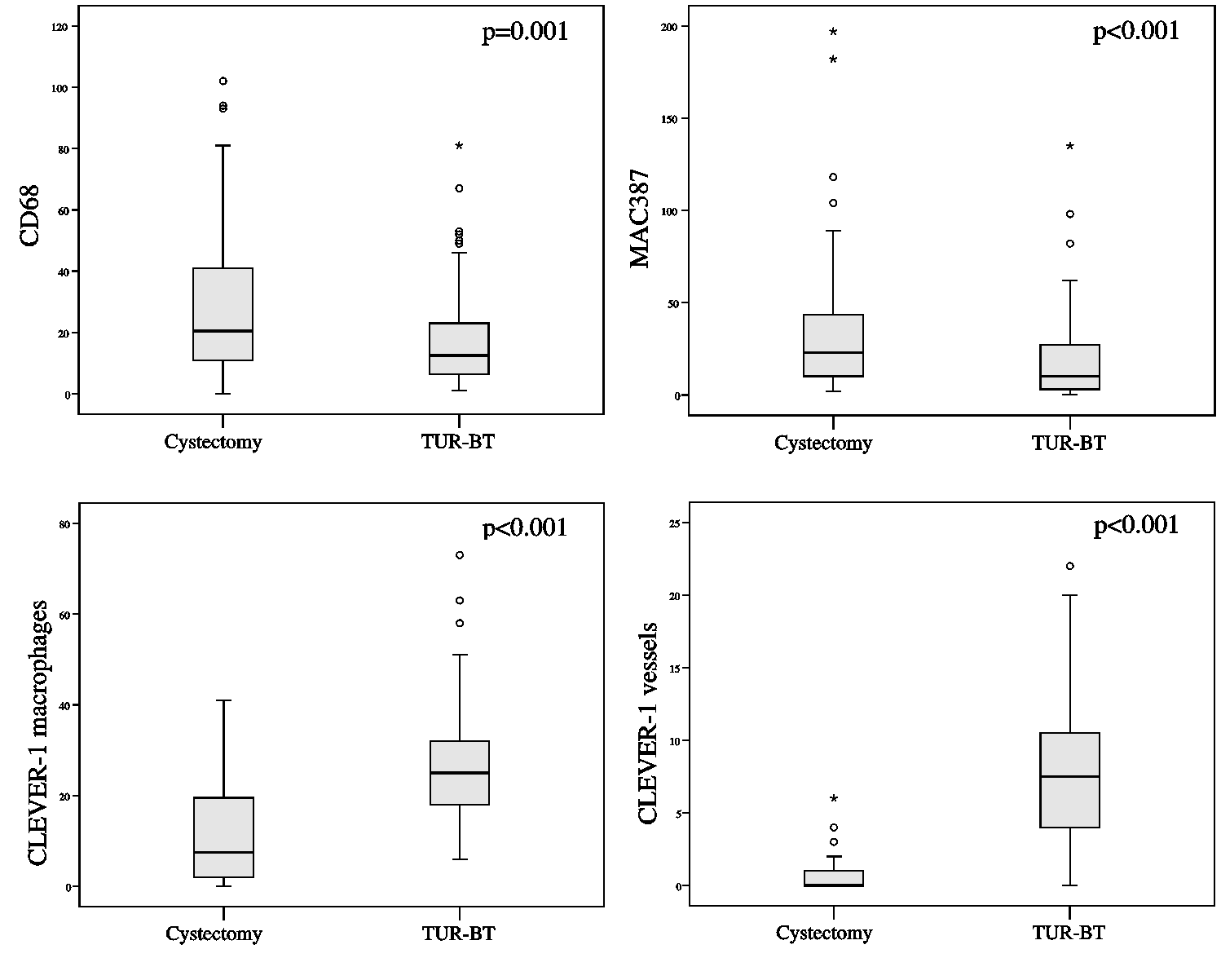

Supplement: S1 Fig — The Mann-Whitney U test was used for pair-wise comparisons. The bottom and top edges of the box indicate the intra-quartile range (IQR), the line inside the box indicates the median value, and the whiskers that extend from each box indicate the range of values that are outside of the intra-quartile range but are closer than or equal to 1.5 times the IQR. Any points that are at a distance of more than 1.5 times the IQR from the box are considered to be outliers. Circles indicate the mild outliers (more than 1.5 times the IQR) and asterisks indicate extreme outliers (more than 3 times the IQR). (TIF) [file pone.0133552.s001.tif]

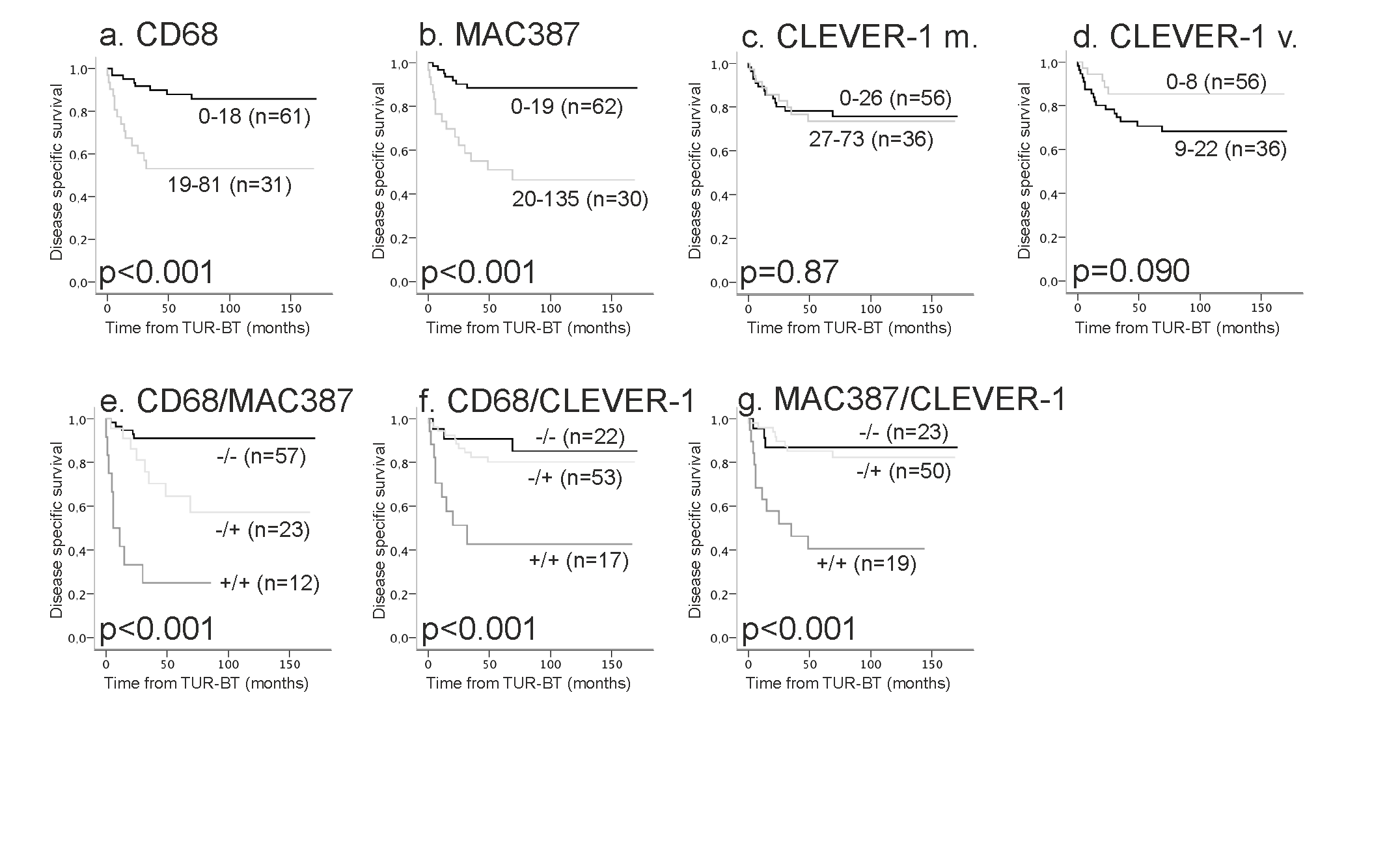

Supplement: S2 Fig — The effect of CD68+ macrophages, MAC387+ macrophages, and CLEVER-1/Stabilin-1+ macrophages/vessels on the DSS in the TUR-BT population (a-d). The association between DSS and the expression of two macrophage markers (e-f). (TIF) [file pone.0133552.s002.tif]

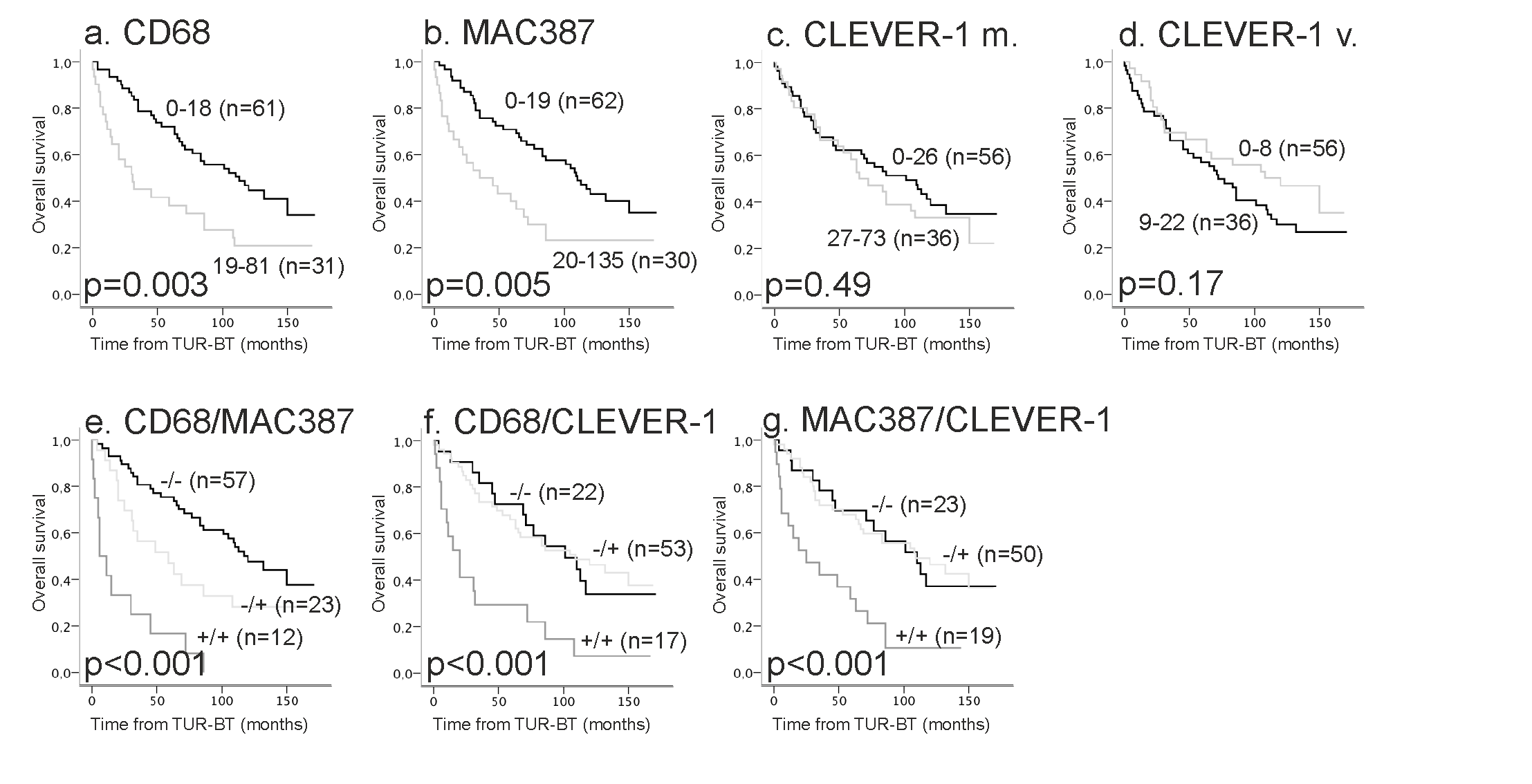

Supplement: S3 Fig — The effect of CD68+ macrophages, MAC387+ macrophages, and CLEVER-1/Stabilin-1+ macrophages/vessels on OS in the TUR-BT population (a-d). The association between OS and the expression of two macrophage markers (e-f). (TIF) [file pone.0133552.s003.tif]

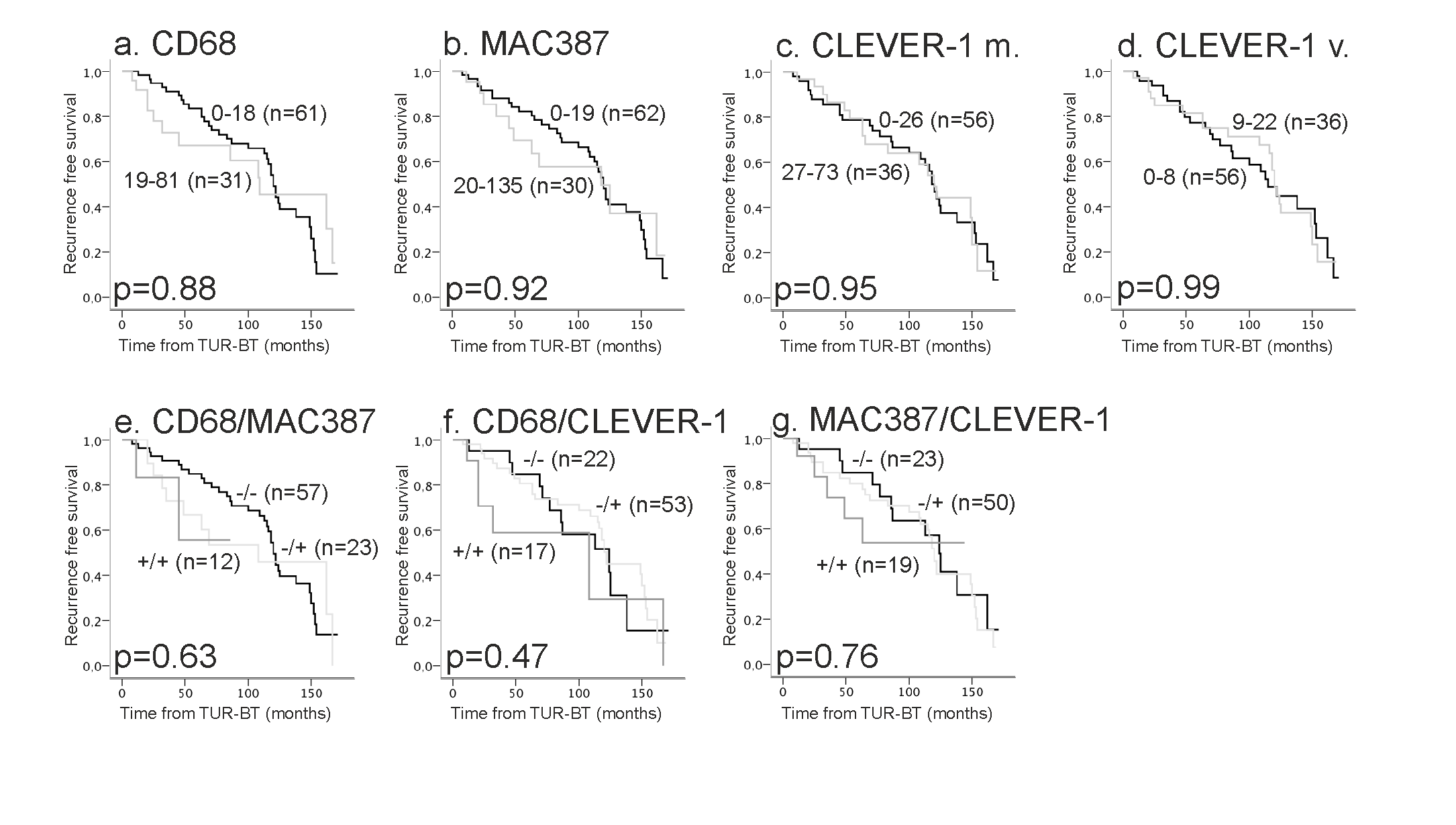

Supplement: S4 Fig — The effect of CD68+ macrophages, MAC387+ macrophages, and CLEVER-1/Stabilin-1+ macrophages/vessels on recurrence in the TUR-BT population (a-d). The association between recurrence and the expression of two macrophage markers (e-f) (TIF) [file pone.0133552.s004.tif]

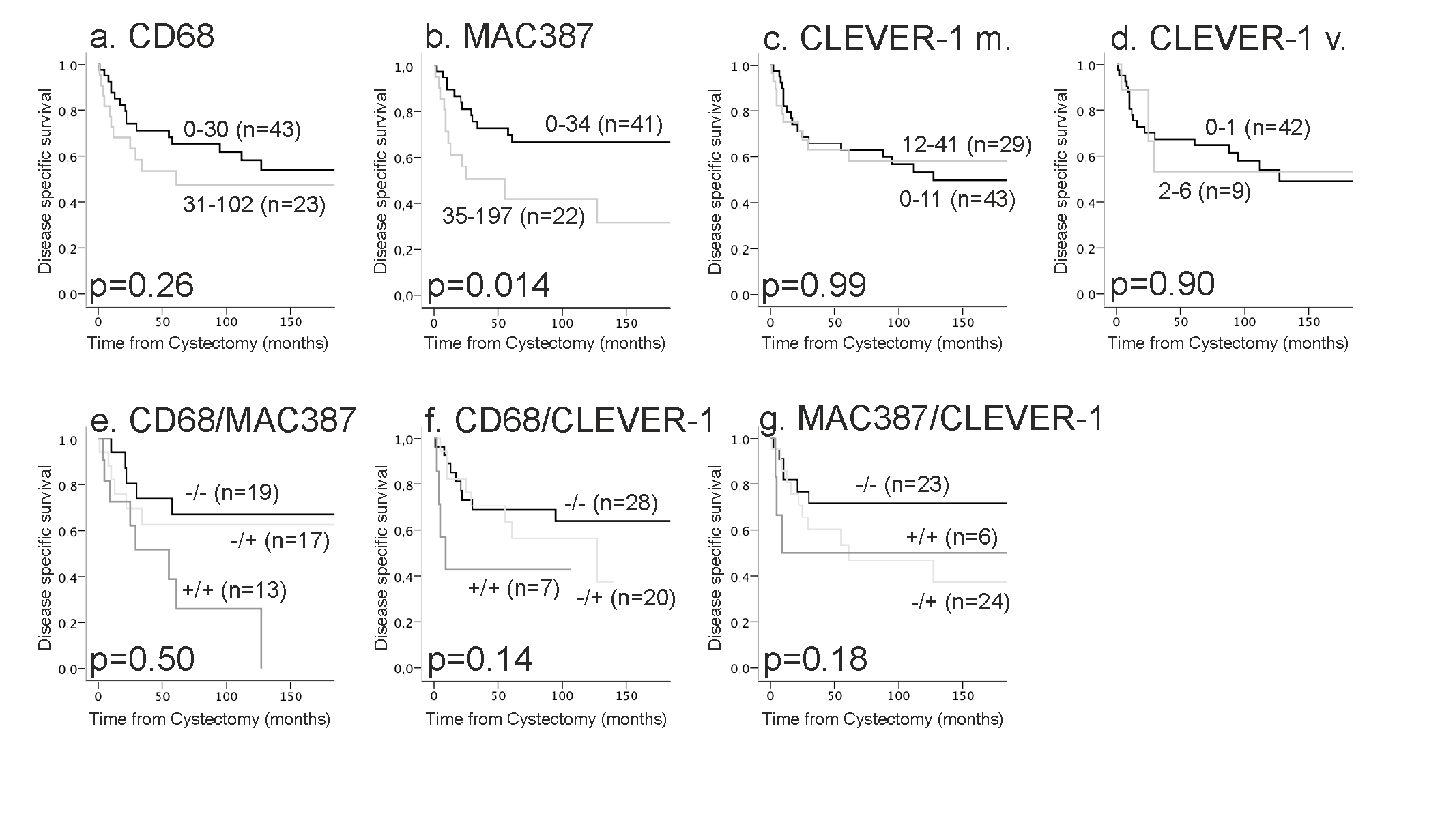

Supplement: S5 Fig — The effect of CD68+ macrophages, MAC387+ macrophages, and CLEVER-1/Stabilin-1+ macrophages/vessels on DSS in the TUR-BT population (a-d). The association between DSS and the expression of two macrophage markers (e-f). (TIF) [file pone.0133552.s005.tif]
