# Supplementary material for: Tumor-Associated Macrophages Provide Significant Prognostic Information in Urothelial Bladder Cancer
Source: PLoS One. 2015 Jul 21;10(7):e0133552. doi: 10.1371/journal.pone.0133552 (PMC4511010; doi:10.1371/journal.pone.0133552)
Supplement: S1 Table — (DOCX) [file pone.0133552.s006.docx]

| Table S1. Characteristics and normalities of scale variables in the whole study cohort. | | | | | |
| --- | --- | --- | --- | --- | --- |
|  | N (%) total 184 | Mean (median) | Range | Skewness | Shapiro-Wilk |
| Age (years) | 184 (100) | 66 (67) | 34-92 | -0.22 | 0.40 |
| Follow-up time (months) | 184 (100) | 75 (72) | 0-213 | 0.37 | <0.001 |
| CD68 | 158 (86) | 23 (17) | 0-102 | 1.72 | <0.001 |
| MAC387 | 155 (84) | 25 (14) | 0.197 | 2.69 | <0.001 |
| CLEVER-1 macroph. | 164 (89) | 20 (19) | 0-73 | 0.80 | <0.001 |
| CLEVER-1 vessels | 143 (78) | 5 (4) | 0-22 | 0.83 | <0.001 |
