# Supplementary material for: Tumor-Associated Macrophages Provide Significant Prognostic Information in Urothelial Bladder Cancer
Source: PLoS One. 2015 Jul 21;10(7):e0133552. doi: 10.1371/journal.pone.0133552 (PMC4511010; doi:10.1371/journal.pone.0133552)
Supplement: S2 Table — (DOCX) [file pone.0133552.s007.docx]

| Table S2. Univariate and multivariate Cox proportional hazards regression analysis of factors affecting DSS on the TUR-BT population. | | | | | | |
| --- | --- | --- | --- | --- | --- | --- |
|  | **Univariate** | | | **Multivariate** | | |
| Variable | **HR** | **95% CI** | **p-value** | **HR** | **95% CI** | **p-value** |
| Grade | | | | | | |
| Low grade | *REF* | | | *REF* | | |
| High grade | 18 | 5.4-62 | <0.001* | 14 | 3.9-50 | <0.001* |
| CD68 | 1.043 | 1.024-1.064 | <0.001* | 1.019 | 0.998-1.041 | 0.075 |
| MAC387 | 1.029 | 1.016-1.042 | <0.001* | 1.011^a^ | 0.996-1.026 | 0.16 |
| CLEVER-1 macroph. | 1.008 | 0.977-1.040 | 0.62 | 0.995^a^ | 0.966-1.024 | 0.73 |
| CLEVER-1 vessels | 0.898 | 0.808-0.998 | 0.046* | 0.989^a^ | 0.892-1.097 | 0.83 |
| CD68/MAC387 | | | | | | |
| CD68/MAC387^-/-^ | *REF* | | | *REF* | | |
| CD68/MAC387^-/+^ | 4.6 | 1.5-14 | 0.007* | 1.6^a^ | 0.48-5.6 | 0.44 |
| CD68/MAC387^+/+^ | 18 | 5.9-54 | <0.001* | 3.4^a^ | 0.95-12 | 0.059 |
| CD68/CLEVER-1 | | | | | | |
| CD68/CLEVER-1^-/-^ | *REF* | | | *REF* | | |
| CD68/CLEVER-1^-/+^ | 1.4 | 0.40-5.3 | 0.58 | 1.7^a^ | 0.46-6.1 | 0.44 |
| CD68/CLEVER-1^+/+^ | 6.3 | 1.7-23 | 0.006* | 2.7^a^ | 0.71-10 | 0.143 |
| MAC387/CLEVER-1 | | | | | | |
| MAC387/CLEVER-1^-/-^ | *REF* | | | *REF* | | |
| MAC387/CLEVER-1^-/+^ | 1.2 | 0.33-4.7 | 0.74 | 0.99^a^ | 0.26-3.7 | 0.98 |
| MAC387/CLEVER-1^+/+^ | 6.3 | 1.8-23 | 0.005* | 2.0^a^ | 0.52-7.3 | 0.32 |
| * Significant p-value  ^a^ Biomarker expressions in a multivariate analyses adjusted for grade. Each biomarker analyzed in a separate multivariate analysis. | | | | | | |
