# Supplementary material for: Tumor-Associated Macrophages Provide Significant Prognostic Information in Urothelial Bladder Cancer
Source: PLoS One. 2015 Jul 21;10(7):e0133552. doi: 10.1371/journal.pone.0133552 (PMC4511010; doi:10.1371/journal.pone.0133552)
Supplement: S3 Table — (DOCX) [file pone.0133552.s008.docx]

| Table S3. Univariate and multivariate Cox proportional hazards regression analysis of factors affecting recurrence on the TUR-BT population. | | | | | | |
| --- | --- | --- | --- | --- | --- | --- |
|  | **Univariate** | | | **Multivariate** | | |
| Variable | **HR** | **95% CI** | **p-value** | **HR** | **95% CI** | **p-value** |
| Grade | | | | | | |
| Low grade | *REF* | | | *REF* | | |
| High grade | 2.0 | 1.0-4.0 | 0.043* | 1.4 | 0.61-3.0 | 0.45 |
| pT-category | | | | | | |
| ≤pT1 | *REF* | | | *REF* | | |
| pT2 | 1.9 | 0.58-6.2 | 0.30 | 1.4 | 0.37-5.8 | 0.59 |
| Age | 1.056 | 1.023-1.090 | 0.001* | 1.052 | 1.018-1.087 | 0.002* |
| CD68 | 1.005 | 0.982-1.029 | 0.68 | 0.996 | 0.972-1.021 | 0.77 |
| MAC387 | 0.998 | 0.980-1.016 | 0.85 | 0.978^a^ | 0.957-1.000 | 0.046* |
| CLEVER-1 macroph. | 1.007 | 0.981-1.033 | 0.61 | 1.002^a^ | 0.977-1.027 | 0.87 |
| CLEVER-1 vessels | 0.997 | 0.931-1.068 | 0.94 | 0.998^a^ | 0.928-1.073 | 0.95 |
| CD68/MAC387 | | | | | | |
| CD68/MAC387^-/-^ | *REF* | | | *REF* | | |
| CD68/MAC387^-/+^ | 1.1 | 0.54-2.2 | 0.81 | 0.88^a^ | 0.40-1.9 | 0.76 |
| CD68/MAC387^+/+^ | 2.0 | 0.46-8.6 | 0.35 | 0.67^a^ | 0.11-4.2 | 0.67 |
| CD68/CLEVER-1 | | | | | | |
| CD68/CLEVER-1^-/-^ | *REF* | | | *REF* | | |
| CD68/CLEVER-1^-/+^ | 0.86 | 0.44-1.7 | 0.67 | 1.0^a^ | 0.52-2.1 | 0.92 |
| CD68/CLEVER-1^+/+^ | 1.5 | 0.55-4.1 | 0.43 | 2.5^a^ | 0.76-8.3 | 0.13 |
| MAC387/CLEVER-1 | | | | | | |
| MAC387/CLEVER-1^-/-^ | *REF* | | | *REF* | | |
| MAC387/CLEVER-1^-/+^ | 1.2 | 0.60-2.3 | 0.63 | 1.6^a^ | 0.81-3.2 | 0.18 |
| MAC387/CLEVER-1^+/+^ | 1.5 | 0.51-4.3 | 0.48 | 0.72^a^ | 0.22-2.4 | 0.59 |
| * Significant p-value  ^a^ Biomarker expressions in a multivariate analyses adjusted for grade and pT-category. Each biomarker analyzed in a separate multivariate analysis. | | | | | | |
