# Supplementary material for: Tumor-Associated Macrophages Provide Significant Prognostic Information in Urothelial Bladder Cancer
Source: PLoS One. 2015 Jul 21;10(7):e0133552. doi: 10.1371/journal.pone.0133552 (PMC4511010; doi:10.1371/journal.pone.0133552)
Supplement: S4 Table — (DOCX) [file pone.0133552.s009.docx]

| Table S4. Univariate and multivariate Cox proportional hazards regression analysis of factors affecting PFS on the TUR-BT population. | | | | | | |
| --- | --- | --- | --- | --- | --- | --- |
|  | **Univariate** | | | **Multivariate** | | |
| Variable | **HR** | **95% CI** | **p-value** | **HR** | **95% CI** | **p-value** |
| Grade | | | | | | |
| Low grade | *REF* | | | *REF* | | |
| High grade | 6.4 | 3.0-14 | <0.001* | 3.2 | 1.3-7.8 | 0.012* |
| pT-category | | | | | | |
| ≤pT1 | *REF* | | | *REF* | | |
| pT2 | 6.4 | 2.9-14 | <0.001* | 2.2 | 0.86-5.6 | 0.102 |
| Age | 1.086 | 1.045-1.129 | <0.001* | 1.055 | 1.015-1.097 | 0.006* |
| CD68 | 1.031 | 1.012-1.050 | 0.001* | 1.005 | 0.983-1.028 | 0.64 |
| MAC387 | 1.022 | 1.010-1.035 | <0.001* | 1.005^a^ | 0.989-1.020 | 0.55 |
| CLEVER-1 macroph. | 1.010 | 0.983-1.038 | 0.48 | 0.999^a^ | 0.974-1.024 | 0.93 |
| CLEVER-1 vessels | 0.910 | 0.837-0.991 | 0.030* | 0.971^a^ | 0.886-1.064 | 0.53 |
| CD68/MAC387 | | | | | | |
| CD68/MAC387^-/-^ | *REF* | | | *REF* | | |
| CD68/MAC387^-/+^ | 1.5 | 0.65-3.5 | 0.34 | 0.79^a^ | 0.31-2.0 | 0.63 |
| CD68/MAC387^+/+^ | 8.8 | 3.7-21 | <0.001* | 1.6^a^ | 0.48-5.0 | 0.46 |
| CD68/CLEVER-1 | | | | | | |
| CD68/CLEVER-1^-/-^ | *REF* | | | *REF* | | |
| CD68/CLEVER-1^-/+^ | 0.98 | 0.41-2.4 | 0.97 | 1.1^a^ | 0.45-2.7 | 0.84 |
| CD68/CLEVER-1^+/+^ | 3.1 | 1.1-8.5 | 0.026* | 1.6^a^ | 0.51-5.2 | 0.41 |
| MAC387/CLEVER-1 | | | | | | |
| MAC387/CLEVER-1^-/-^ | *REF* | | | *REF* | | |
| MAC387/CLEVER-1^-/+^ | 1.1 | 0.43-2.6 | 0.91 | 1.3a | 0.51-3.1 | 0.62 |
| MAC387/CLEVER-1^+/+^ | 3.8 | 1.4-10 | 0.007* | 1.4^a^ | 0.49-4.1 | 0.52 |
| * Significant p-value  ^a^ Biomarker expressions in a multivariate analyses adjusted for grade, pT-category and age. Each biomarker analyzed in a separate multivariate analysis. | | | | | | |
