# Supplementary material for: Tumor-Associated Macrophages Provide Significant Prognostic Information in Urothelial Bladder Cancer
Source: PLoS One. 2015 Jul 21;10(7):e0133552. doi: 10.1371/journal.pone.0133552 (PMC4511010; doi:10.1371/journal.pone.0133552)
Supplement: S5 Table — (DOCX) [file pone.0133552.s010.docx]

| Table S5. Univariate and multivariate Cox proportional hazards regression analysis of factors affecting DSS on the RC population. | | | | | | |
| --- | --- | --- | --- | --- | --- | --- |
|  | **Univariate** | | | **Multivariate** | | |
| Variable | **HR** | **95% CI** | **p-value** | **HR** | **95% CI** | **p-value** |
| Grade | | | | | | |
| Low grade | *REF* | | | *REF* | | |
| High grade | 2.4 | 0.93-6.1 | 0.072 | 2.3 | 0.65-8.3 | 0.19 |
| pT-category | | | | | | |
| ≤pT1 | *REF* | | | *REF* | | |
| pT2 | 4.1 | 1.3-13 | 0.017* | 4.0 | 0.98-16 | 0.053 |
| pT3 | 10 | 3.4-31 | <0.001* | 8.8 | 2.4-33 | 0.001* |
| pT4 | 14 | 4.4-47.3 | <0.001* | 11 | 2.7-44 | 0.001* |
| CD68 | 1.004 | 0.991-1.017 | 0.57 | 0.994 | 0.987-1.011 | 0.51 |
| MAC387 | 1.008 | 1.001-1.016 | 0.032* | 1.003^a^ | 0.993-1.012 | 0.59 |
| CLEVER-1 macroph. | 1.020 | 0.984-1.058 | 0.29 | 1.017^a^ | 0.982-1.054 | 0.33 |
| CLEVER-1 vessels | 1.105 | 0.765-1.595 | 0.60 | 1.222^a^ | 0.857-1.741 | 0.27 |
| CD68/MAC387 | | | | | | |
| CD68/MAC387^-/-^ | *REF* | | | *REF* | | |
| CD68/MAC387^-/+^ | 1.5 | 0.45-4.9 | 0.52 | 1.6^a^ | 0.41-6.1 | 0.51 |
| CD68/MAC387^+/+^ | 3.5 | 1.1-11 | 0.029* | 2.2^a^ | 0.65-7.5 | 0.21 |
| CD68/CLEVER-1 | | | | | | |
| CD68/CLEVER-1^-/-^ | *REF* | | | *REF* | | |
| CD68/CLEVER-1^-/+^ | 1.5 | 0.56-3.8 | 0.45 | 0.73^a^ | 0.27-2.0 | 0.55 |
| CD68/CLEVER-1^+/+^ | 3.2 | 0.96-10 | 0.060 | 1.5^a^ | 0.43-5.2 | 0.53 |
| MAC387/CLEVER-1 | | | | | | |
| MAC387/CLEVER-1^-/-^ | *REF* | | | *REF* | | |
| MAC387/CLEVER-1^-/+^ | 2.2 | 0.80-5.9 | 0.13 | 0.88^a^ | 0.29-2.7 | 0.82 |
| MAC387/CLEVER-1^+/+^ | 3.0 | 0.74-12 | 0.13 | 3.9^a^ | 0.80-19 | 0.092 |
| * Significant p-value  ^a^ Biomarker expressions in a multivariate analyses adjusted for grade, pT-category and age. Each biomarker analyzed in a separate multivariate analysis. | | | | | | |
